# Paraneoplastic anti-N-methyl-D-aspartate receptor encephalitis associated with small cell lung cancer and cytotoxic T-cell-mediated pathology: Case report

**DOI:** 10.3389/fimmu.2022.952868

**Published:** 2022-08-19

**Authors:** Haruna Akanuma, Takahiro Iizuka, Dan Abe, Kenji Yoshida, Nozomu Matsuda, Kotaro Sugimoto, Yuko Hashimoto, Kazuaki Kanai

**Affiliations:** ^1^ Department of Neurology, Fukushima Medical University School of Medicine, Fukushima, Japan; ^2^ Department of Neurology, Kitasato University School of Medicine, Sagamihara, Japan; ^3^ Department of Pathology, Fukushima Medical University School of Medicine, Fukushima, Japan

**Keywords:** paraneoplastic encephalitis, small cell lung cancer, Anti-N-methyl-d-aspartate receptor antibodies, pathology, cytotoxic T-cells

## Abstract

Anti-N-methyl-D-aspartate receptor (NMDAR) antibody encephalitis is caused by a reversible inhibition of ion channel actions by autoantibodies and is associated with a relatively good prognosis. Pathological findings of NMDAR encephalitis usually do not show neurophagorous nodules, but rare or mild inflammatory infiltration. We report a patient of small cell lung cancer (SCLC)-related paraneoplastic encephalitis with NMDAR antibodies, a cytotoxic T-cell-mediated pathology of the brain, and a rapid clinical course. This case highlights that the neuropathological diversity of NMDAR encephalitis may be even broader than previously thought and that NMDAR antibodies may also be found in various pathological conditions with a vigorous immune response.

## Introduction

Paraneoplastic neurological syndromes are classically associated with autoantibodies against neuronal intracellular antigens. Graus et al. recently proposed that such onconeural antibodies should be classified as high-risk (>70% associated with cancer) and intermediate-risk (>30%–70% associated with cancer) (1). Cytotoxic T-cell infiltration of the nervous system is usually observed in classical paraneoplastic syndromes with high-risk antibodies to intracellular antigens (2). On the other, the anti-N-methyl-D-aspartate receptor (NMDAR) antibody is to cell-surface antigen and categorized in the intermediate-risk group. In patients with anti-NMDAR encephalitis (NMDARE), nerve tissue destruction is usually mild, neurological manifestations are likely caused by reversible inhibition of ion channel actions by autoantibodies, and infiltration of cytotoxic T cells is rare (3).

Here, we report a patient with paraneoplastic encephalitis associated with small cell lung cancer (SCLC) and NMDAR antibodies with a cytotoxic T-cell immune response and atypically rapid clinical course.

## Case presentation

A 72-year-old woman presented to our hospital with increasingly frequent headaches over 2 months, hallucinations, and lethargy; for example, she became irritable and began to say that there were people who were not really there. She had a history of diabetes, atrial fibrillation, and 55-pack-year smoking.

**Figure 1:**
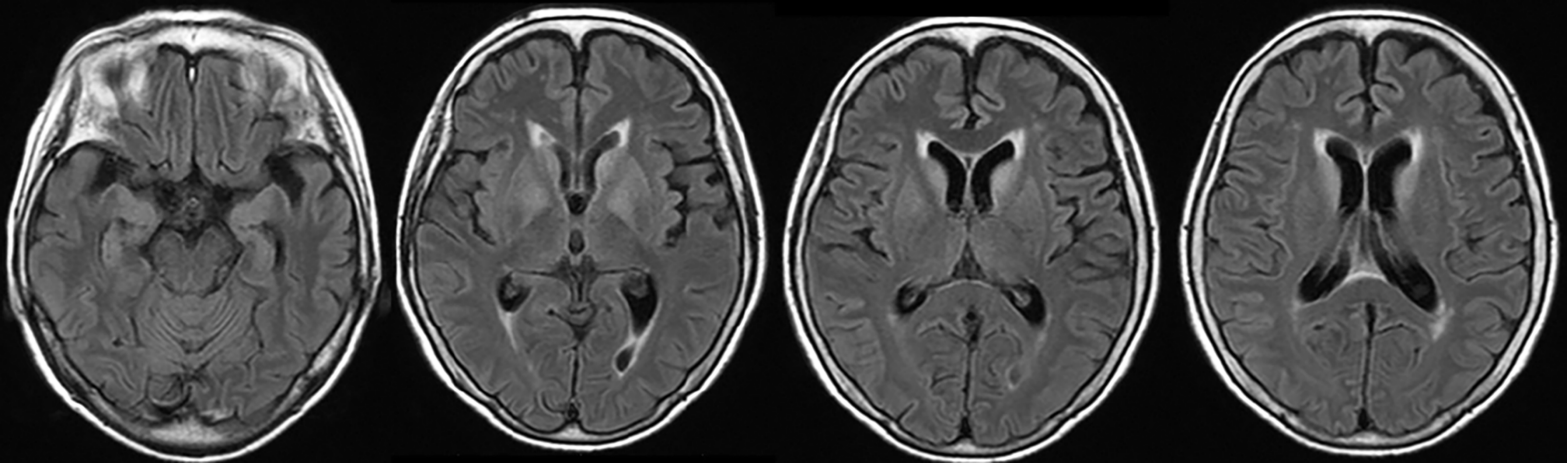
Brain MRI showed symmetric increased fluid-attenuated inversion recovery signals in the basal ganglia and medial temporal lobes.

**Figure 2:**
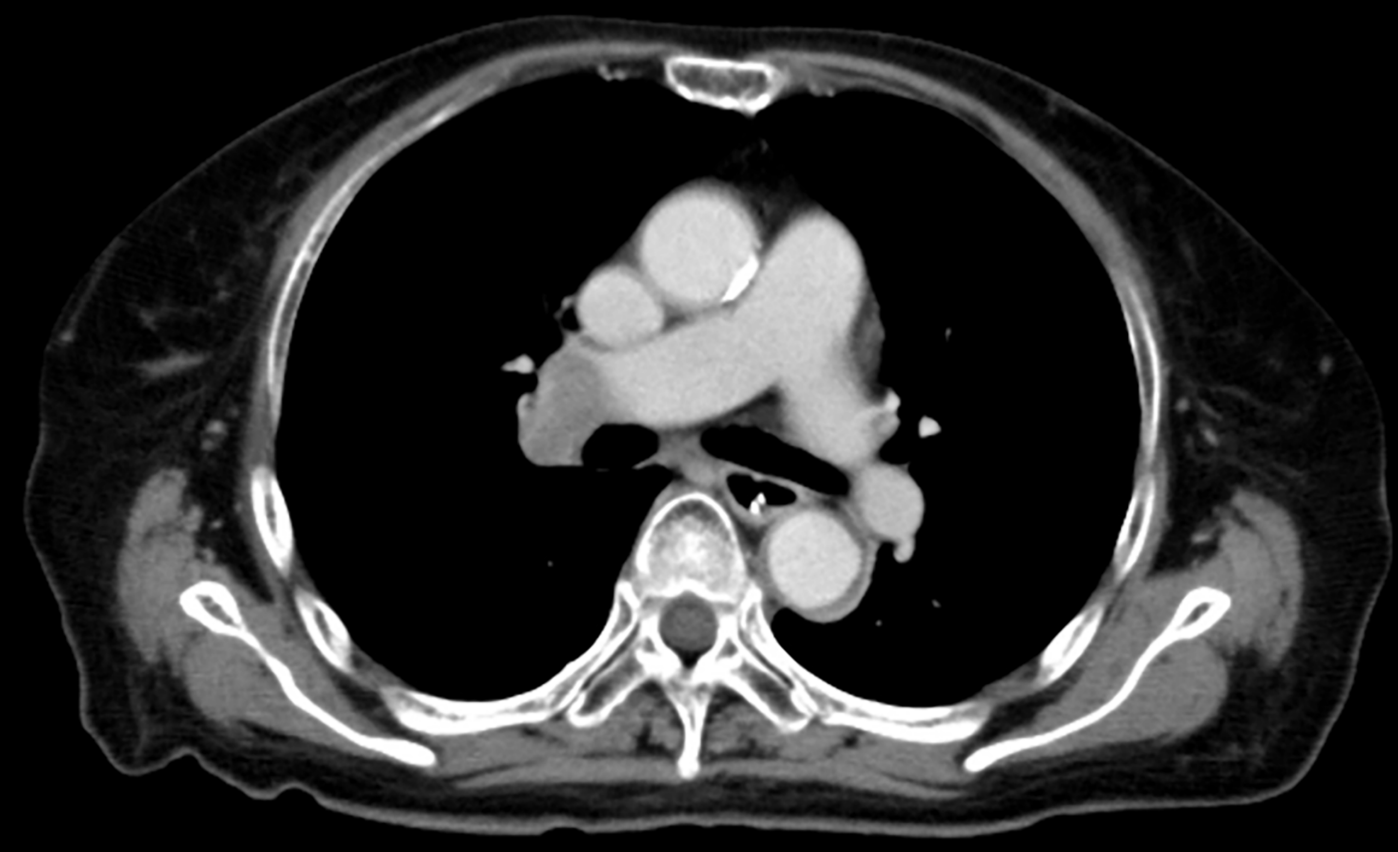
Full-body CT revealed a mass in the right hilar region.

On admission, she was afebrile and vital signs were unremarkable. Neurological examination revealed impaired consciousness (Glasgow Coma Scale E3V3M6), right ptosis, and paratonic and nuchal rigidity. There was no abnormality in the blood test: red blood cells 4.25 × 10^6^/μl, white blood cells 5,700/μl, platelet 16.0 × 10^6^/μl, blood glucose level 142 mg/dl, aspartate aminotransferase 17 IU/l, alanine aminotransferase 13 IU/l, blood urea nitrogen 9 mg/dl, creatinine 0.55 mg/dl, sodium concentration 135 mEq/l, potassium concentration 3.6 mEq/l, and C-reactive protein 0.19 mg/dl. Cerebrospinal fluid (CSF) examination revealed 25 cells/μl (mononucleated 96%), protein 154 mg/dl, glucose 89 mg/dl, positive CSF-restricted oligoclonal bands, and a high IgG index (1.05). Cytologic studies of CSF showed no malignant cells. Her serum and CSF were negative for all classical (intracellular) paraneoplastic, glial fibrillary acidic protein, and neuronal surface antibodies (including gamma-aminobutyric acid B and α-amino-3-hydroxy-5-methyl-4-isoxazolepropionic acid receptor antibodies), except for NMDAR antibodies, which were detected in the CSF. These autoantibodies were identified by well-established rat brain immunohistochemistry (IHC) and cell-based assays (CBA) in Dalmau’s Laboratory (Barcelona) and Kitasato University (Japan). Additionally, according to a commercial immunoblotting assay, no serum anti-neuronal antibodies were identified for the following 12 antigens: Hu. Ri, Yo, SOX1, CV2, amphiphysin, Ma2/Ta, Zic4, recoverin, titin, GAD65, and Tr/DNER. Brain MRI showed symmetric increased fluid-attenuated inversion recovery signals in the basal ganglia and medial temporal lobes (**Figure 1**). Full-body CT revealed a mass in the right hilar region, consistent with a diagnosis of SCLC (**Figure 2**). Fluorine-18 fluorodeoxyglucose [(18)F-FDG]-positron emission tomography (PET) revealed increased uptake of tracer in the right hilum but no apparent distant metastasis. An electroencephalogram showed unremarkable results.

We strongly suspected paraneoplastic encephalitis associated with SCLC on the basis of the above findings and therefore instituted high-dose methylprednisolone (1,000 mg daily intravenously for 3 days) from day 18 of admission with no improvement. On day 23, the patient had a cardiopulmonary arrest. Cardiopulmonary resuscitation was performed for a while, but the family wanted to stop it on the way. The patient died 9 h later after sudden change.

**Figure 3:**
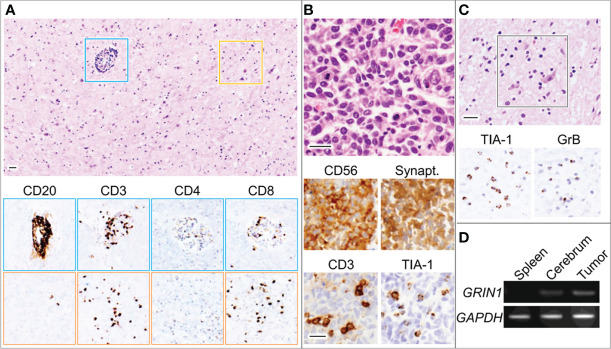
Microscopic images for the globus pallidus **(A, C)** and lung tumor **(B)**. Blue and orange squares indicate perivascular regions and parenchyma with neurons, respectively. Bars, 50 μm. **(D)** RT-PCR using total RNA derived from the indicated organs of the patient **(D)**. GAPDH, glyceraldehyde 3-phosphate dehydrogenase; GrB, granzyme B; GRIN1, glutamate ionotropic receptor N-methyl-D-aspartate type subunit 1; Synapt, Synaptophysin; TIA-1, T-cell intracytoplasmic antigen-1.

Postmortem examination revealed infiltration of the CNS with small mononuclear cells, most prominently in the limbic system and brainstem, including the respiratory center, moderately in the cerebral cortex and lumbar cord, but not in the cerebellum. Activated neuronophagic cytotoxic T cells (CTL) that were positive for CD3, CD8, TIA-1, and granzyme B were diffusely spread throughout the brain parenchyma, whereas CD20-positive B cells were localized in the perivascular regions (**Figures 3A, C**). No neutrophils, eosinophils, or foreign body-type giant cells were identified. Thus, our patient’s neurologic manifestations were presumably caused by autoimmune-mediated encephalitis, dominantly triggered by mechanisms of cellular immunity with CTL infiltration. The right lung tumor was diagnosed pathologically as a neuroendocrine marker-positive SCLC with scant cytoplasm and salt-and-pepper nuclei (**Figure 3B**). CTL were identified infiltrating the stroma of the carcinoma, suggesting that it had triggered the T-cell-mediated response responsible for the paraneoplastic encephalitis. Furthermore, we conducted quantitative polymerase chain reaction (qPCR) which revealed GluN1 gene expression in the SCLC tissue (**Figure 3D**). Although we could not conduct IHC in the tumor tissue, mRNA was extracted from the FFPE specimen using TRIzol reagent following a standard protocol. No other lesions suggestive of malignant tumors were found throughout the body, including the ovaries.

## Discussion

This case shows that NMDARE associated with SCLC may have an atypical clinical course and pathological findings (extensive infiltration of CTL throughout the CNS), similar to those in paraneoplastic encephalitis with high-risk autoantibodies.

NMDARE is caused by a reversible inhibition of ion channel actions by autoantibodies and is associated with good prognosis (2). The mortality rate of typical NMDARE is usually low; therefore, only a small number of autopsy cases of NMDARE have been reported. In such reports, it was revealed that the pathological findings of NMDARE were characteristically unaccompanied by neuronophagic nodules and with rare or mild inflammatory infiltration (4, 5). On the other, classical SCLC-associated paraneoplastic encephalitis/encephalomyelitis with high-risk autoantibodies is known to be accompanied by CTL-mediated pathology of the brain, and its clinical course is usually severer than of NMDARE.

To date, encephalitis complicating SCLC with NMDAR antibodies and with a poor prognosis has also been reported (6–15) (**Table 1**). There were only two cases where pathological autopsy was performed and both died in a rapid course. One case was not positive for TIA-1 and neuronal loss was mild, but T-cell infiltration was observed not only in the perivascular but also in the parenchymal (12).

**Table 1:** Clinical features of cases of encephalitis complicating SCLC with NMDAR antibodies.

Nr	Onset Age	Sex	Clinical Symptoms	seizure	hypo-ventilation	IVM	Duration(M)	Pathological Examination	T-cell infiltration	TIA-1-positivity	Ref.
			psychosis					Findings			
1	62	F	+	+	+	+	1	NA	NA	NA	6
2	61	M	+	+	-	+	12	Gliosis (hippocampus, amygdala, and claustrum)	NA	NA	7
3	NA	NA	NA	NA	NA	NA	NA	NA	NA	NA	8
4	76	M	+	-	+	-	1	NA	NA	NA	9
5	66	F	+	-	-	-	>12*	NA	NA	NA	10
6	58	M	+	-	+	+	NA	NA	NA	NA	11
7	76	M	+	-	-	-	0.5	Mild Neuronal loss and gliosis (hippocampus)	+	-	12
8	62	M	+	+	-	-	>15*	NA	NA	NA	13
9	67	F	+	-	-	+	57	NA	NA	NA	14
9	66	M	+	+	-	-	1	NA	NA	NA	14
9	62	F	+	+	-	+	2	NA	NA	NA	14
10	NA	NA	NA	NA	NA	NA	NA	NA	NA	NA	15

*: Nr 5, 8 is alive at the time of reporting.

Nr, Nummer; IVM, Involuntary Movement; TIA-1, T-cell intracytoplasmic antigen-1; Ref, References. NA, Not Available.

In our case, the coexistence of obvious CTL-mediated pathology of the brain and only NMDAR antibodies, which is a disease-specific one, was a characteristic pathological finding. So far, similar autoimmune diseases such as type 1 diabetes and anti-PIT-1 hypophysitis have been reported, in which disease-specific autoantibodies and CTL pathology coexist. In such diseases, it has been shown that antigen-presenting cells with highly immunogenic antigens may induce both CTL activation and autoantibody production through activation of CD4-positive T cells (16). Similarly, in our case, SCLC may cause both CTL pathology in the brain and anti-NMDAR antibody positivity reflecting a vigorous immune response through NMDAR expression. A rapidly progressive clinical course of our case might also reflect such underlying vigorous immune response.

Furthermore, Zrzavy et al. recently reported that the topographic distribution of inflammation in patients with NMDAR encephalitis reflects the clinical course (5). Although we did not conduct an IHC to NMDAR of the brain tissue of this patient and topographic change of NMDAR distribution was unclarified, we found that the areas with maximal inflammation were limbic system and brainstem including the respiratory center, which correlated with the clinical presentation of sudden cardiopulmonary arrest, supporting the conclusion by Zrzavy et al. Also, we considered that the abnormalities in MRI were due to inflammation caused by CTL, because pathological examination showed strong inflammation of the limbic system as mentioned earlier.

On the other hand, it might be possible that SCLC in itself may cause CTL-mediated responses in the brain and accidental coexistence of the NMDAR antibody. Raspotnig et al. reported that four of 14 patients with SCLC and paraneoplastic encephalitis had no detectable onconeural autoantibodies (17). However, Bastiaansen et al. had shown that there were no false positives if anti-NMDAR antibodies were detected in both CBA and IHC in the CSF (15). We considered that it was not a coincidence that anti-NMDAR antibodies were detected in this patient, because it was not only positive for CBA in CSF but also positive for IHC in CSF, although this case was clinically atypical. In addition, we confirmed the expression of the NR1 subunit in samples of SCLC by qPCR. Several previous reports found that the NR1 subunit was expressed in tumor tissue and the findings had been thought to suggest an association between anti-NMDAR antibodies and tumor (6–8, 14). It is well known that NMDARE is often accompanied by hypoventilation in its clinical course (18). Two of three reported patients with SCLC-related encephalitis with NMDAR antibodies and pathologically proven fatal encephalitis had aggressive clinical courses and poor prognoses (7, 12). We conjecture that hypoventilation actions of the antibody may be responsible for the shorter survival of patients with SCLC-related encephalitis with the NMDAR antibody, including our case, although the copresence of undetected high-risk antibodies might also contribute to a severe clinical course.

This report is a single and retrospective case study, and the possibility of contribution of undetected paraneoplastic antibodies is not fully excluded. Nevertheless, this case highlights that neuropathological diversity may be even broader than previously thought. We think that the accumulation of further pathological examinations will be needed.

## Data availability statement

The original contributions presented in the study are included in the article/supplementary material. Further inquiries can be directed to the corresponding author.

## Ethics statement

This retrospective observational single case report was approved by the Institutional Review Board of Kitasato University (B20-280), and a written consent was obtained from the patient and family. Written informed consent was obtained from the individual(s) for the publication of any potentially identifiable images or data included in this article.

## Author contributions

HA and KK contributed to the drafting of the entire manuscript and the preparation of the figures. TI contributed to the analysis of data and the drafting of a significant portion of manuscript. KS and YH contributed to the analysis of data and the preparation of the figures. DA, KY, and NM contributed the acquisition of data. All translations into English were done by the authors. All authors contributed to the article and approved the submitted version.

## Acknowledgments

We particularly thank Professor Josep Dalmau of the Institut d´Investigació Biomèdica August Pi i Sunyer (IDIBAPS), Barcelona, Spain, for extensive studies on antibodies against neuronal surfaces and synaptic proteins at the Dalmau Laboratory. We also thank Dr. Trish Reynolds, MBBS, FRACP, from Edanz (https://jp.edanz.com/ac) for editing a draft of this manuscript.

## Conflict of interest

The authors declare that the research was conducted in the absence of any commercial or financial relationships that could be construed as a potential conflict of interest.

## Publisher’s note

All claims expressed in this article are solely those of the authors and do not necessarily represent those of their affiliated organizations, or those of the publisher, the editors and the reviewers. Any product that may be evaluated in this article, or claim that may be made by its manufacturer, is not guaranteed or endorsed by the publisher.
